# Exploring Teachers’ Resource Utilization Practices and Beliefs in Mathematics Education: A Cross-National Study on Reasoning and Proving

**DOI:** 10.1007/s10763-025-10577-4

**Published:** 2025-05-14

**Authors:** Iveta Kohanová, Mária Slavíčková, Samuel Rosa, Benedetto Di Paola, Jakub Michal, Erdinç Çakıroğlu

**Affiliations:** 1 Department of Teacher Education, Norwegian University of Science and Technology, Sverres Gate 12, N-7491 Trondheim, Norway; 2 Faculty of Mathematics, Physics and Informatics, Comenius University in Bratislava, Mlynská Dolina, 842 48 Bratislava, Slovakia; 3 Department of Mathematics and Computer Science, University of Palermo, Via Archirafi 34, 90123 Palermo, Italy; 4 Faculty of Education, Charles University, M. Rettigové 4, 116 39 Prague, Czech Republic; 5 Faculty of Education, Middle East Technical University, 06800 Ankara, Turkey

**Keywords:** Mathematics teachers, Reasoning and proving, Resources, Statistical correlation

## Abstract

This study delves into resource utilization for teaching reasoning and proof (R&P) among mathematics teachers across five European countries. Employing an online questionnaire, the research gathers data on resource use, beliefs about R&P importance, teachers’ self-reported confidence in R&P instruction, and demographic factors. Through statistical analyses, including correlation, logistic regression, and chi-squared tests, the study explores relationships between teachers’ resource use, beliefs, self-reported confidence in R&P instruction, and demographics. Results unveil a significant yet moderate association between resource utilization for lesson preparation and R&P instruction, with variations noted among resource types. While certain resources like professional periodicals/journals and online professional platforms display moderate correlations, others like general websites exhibit weaker ones. Moreover, the study identifies a significant but weak association between resource use, beliefs about R&P, and teachers’ self-reported confidence in R&P instruction. The country emerged as a prominent determinant of resource utilization patterns, with notable consistency observed in the use of textbooks and self-created materials across national contexts. These findings underscore the nuanced nature of mathematics teachers’ resource practices, emphasizing the need to consider diverse factors, such as level of instruction or cultural context, in understanding R&P instructional practices. The implications of these findings for educational practice, policy, and future research are discussed.

## Introduction

In times of educational change, well-designed resources and sufficient resourcing are crucial for math curriculum reforms. In the latest International Commission on Mathematical Instruction (ICMI) study, Golding (2023) highlights the risks of inadequate resourcing or rushed resource design, advocating for textual, manipulative, and digital resources to enhance student engagement and facilitate teachers'implementation of curriculum reforms. However, she highlights a research gap concerning the potential advantages and limitations of digital materials. With the rise of new digital technologies and the increased use of them, especially in the context of the COVID-19 and post-COVID era, there is a pressing need for extensive research into their impact on educational systems (e.g., Engelbrecht et al., 2020; Vithal & Shimizu, 2023). On the other hand, teachers are essential for curriculum reforms as resources alone cannot alter teaching or learning practices (Rezat et al., 2021). Effective innovations necessitate supporting teachers and students in co- and re-designing curriculum materials to align with reform goals and individual needs (Rezat et al., 2021).

Recent curricular changes in European countries like Norway, Slovakia, and Italy emphasize the growing significance of reasoning and proving (R&P) in mathematics lessons. However, teaching R&P is challenging (Buchbinder & McCrone, 2022; Herbert & Williams, 2023), particularly at primary and lower secondary levels. Researchers have proposed explanations (e.g., Cai & Cirillo, 2014; Stylianides, 2008b) and strategies to enhance R&P instruction (e.g., Davidson et al., 2019; Ellis et al., 2019; Nardi & Knuth, 2017). In the 19th ICMI study, Lin et al. (2012) highlighted that the existing literature focuses more on aspects of teachers'knowledge concerning teaching R&P, compared to their practices and beliefs on this topic. They recommended further investigations focusing on “teachers’ beliefs and practices and the interrelationship amongst knowledge, beliefs, and practice in the teaching of proofs and proving” (p. 342). Our study, spanning five European countries, examines teachers’ resource utilization and its relation to their beliefs and practices regarding R&P instruction, addressing this gap.

The study was conducted as part of the European project MaTeK, involving partners from the Czech Republic, Italy, Norway, Slovakia, and Turkey. It served as a context and needs analysis for the consortium’s intervention study. The intervention aimed at enhancing pre-service mathematics teachers’ lesson design capacity related to R&P (Slavíčková et al., 2022). According to project consortium members, lesson planning with a focus on R&P was also a challenge for pre-service mathematics teachers at their institutions across all five countries, a phenomenon observed by several researchers (e.g., Buchbinder & McCrone, 2023; Stylianides et al., 2013).

Insights gained from cross-national studies, particularly those involving diverse countries like the Czech Republic, Italy, Norway, Slovakia, and Turkey, can shed light on mathematics teachers’ resource utilization practices across varied educational contexts. By studying these countries, which are rarely examined together, we can uncover common patterns, unexpected phenomena and practices. This valuable information supports mathematics education researchers and educators in their efforts to globally support teachers (Remillard, 2019).

## Theoretical Framing

In this study, we use the theoretical frames of (a) the Documentational Approach to Didactics, (b) Resources, and (c) Reasoning and proving, for our purpose of exploring teachers’ practices in utilizing various resources for mathematics lesson planning and for teaching R&P.

### The Documentational Approach to Didactics

The documentational approach to didactics (DAD) is a theory in mathematics education that focuses on understanding teachers’ professional development through their interactions with the resources they use and design (Shao et al., 2023). This framework offers a valuable lens through which to analyse a teacher’s activity and engagement with resources within a teaching situation (e.g., when designing a lesson plan with a specific learning goal for a particular grade). Teachers actively search for resources—anything that can re-source their practice (Adler, 2000), but they also encounter useful resources unexpectedly, for example, through unplanned informal discussions with colleagues (Gueudet, 2017). They integrate these resources with their knowledge, adapt or create new ones, and use them in their teaching. This activity, known as *documentation work*, is central to teachers’ professional practice. Through it, teachers develop *a document*, consisting of a set of resources and related *utilization schemes* for a particular *class of situations* (context in which resources are utilized). Furthermore, a utilization scheme encompasses an *aim* and *rules of action* (consistent patterns in how resources are used) that are governed by *operational invariants* (elements of the teacher’s beliefs and knowledge that both influence and result from their teaching practices) (Gueudet, 2017; Gueudet & Trouche, 2009; Pocalana & Robutti, 2024). The process of development of a document is called *a documentational genesis*.

For example, a mathematics teacher engaged in the class of situations of lesson preparation aims to design a review lesson before a test. They might start by consulting a textbook to identify key concepts and procedures and then select essential exercises from the textbook or past worksheets, adjusting task contexts and numbers using AI. Before reusing the task list with another class, they refine it based on pupils’ needs. Over time, this document evolves through different contexts. A typical rule of action might be to include a few tasks from the previous year’s test, guided by the operational invariant that *“exemplary items help pupils better understand what to expect.”* In this case, the class of situations of lesson preparation is associated with the general aim of preparing a review lesson, which, in turn, can encompass multiple documents corresponding to different sub-aims. For instance, a content-specific sub-aim, such as reviewing fractions, would lead to a distinct document tailored to that mathematical content (Gueudet, 2017).

The *resources system* encompasses all resources a teacher uses, structured according to different aims (Gueudet, 2017; Gueudet & Trouche, 2011). While a specific document serves a particular aim (e.g., preparing a review lesson on fractions), the resources system integrates resources across various aims. For instance, beyond designing review lessons, the same teacher may also prepare a lesson introducing the concept of fractions. This aim requires different resources, perhaps a combination of a textbook explanation, hands-on manipulatives like fraction circles, and an interactive online simulation. The operational invariant guiding this document could be that *“pupils grasp fractions better through visual and tactile experiences.”* Even though the documents for review lessons and concept introduction serve different aims, they both emerge from the teacher’s broader resources system, which integrates textbooks, past worksheets, tests, digital tools, and instructional strategies across multiple teaching situations. Thus, while the documents system consists of structured sets of documents associated with different aims, the resources system includes all the resources that contribute to these documents, regardless of their specific scheme of utilization in a particular aim.

DAD also emphasizes how the documentational genesis occurs within individual and collective utilization schemes, involving the dimensions of instrumentation (how resources are adapted) and instrumentalization (how these resources are used as instruments for teaching) (Gueudet & Trouche, 2011). The documentational approach to didactics has evolved over time, both theoretically and methodologically, and has been used to study teachers’ documentation work and adaptations to new teaching resources (Trouche et al., 2020).

### Resources

The term *resource* carries a range of meanings in both everyday and educational contexts (Ruthven, 2013). In our study, a *resource* refers to any asset that teachers utilize to design and enact instruction. In the phase of lesson design, resources support teachers’ decisions about mathematical content (what should be taught and in what order), instructional design (how and why it should be taught), and enacting the teaching (Gustafsson et al., 2024). Pepin and Gueudet (2018) distinguish between material and nonmaterial resources, where the latter encompasses *social resources* (i.e., direct or online conversations with colleagues) and *cognitive resources* (i.e., mathematical and teaching-related concepts, routines, and frameworks teachers use and/or develop). Regarding material resources, they define *curriculum resources* as “all the material resources that are developed and used by teachers and students in their interaction with mathematics in/for teaching and learning, inside and outside the classroom” (p. 1). In other words, curriculum resources are intended to be aligned with the mathematics curriculum. Similarly, Remillard (2019) views curriculum resources as those “designed to support a program of instruction and student learning over time” (p. 179). However, Remillard considers curriculum resources as a subset of *instructional resources*, a broader category encompassing “artifacts provided to, appropriated by, or generated by teachers to guide or support classroom instruction” (p. 179), including those that are not curricular. Pepin and Kock (2021) introduced the term *general resources* to describe non-curricular materials undergraduates employ in challenge-based learning. Applied to teachers’ resource use, we interpret general resources as those not originally intended for curriculum enactment, such as YouTube or Wikipedia, aligning with Remillard’s broader category of instructional resources. Moreover, Remillard (2019), drawing on the DAD (Trouche et al., 2020), noted a special type of curriculum resource, *documents*. As mentioned earlier, documents are products of teachers’ curriculum design, representing resources shaped by the teacher’s utilization schemes. In other words, documents are teacher-created curriculum resources that might be based on various types of resources (social, cognitive, curriculum, and general), evolve with experience, reflecting beliefs, and choices while adapting to different classroom situations.

**Fig. 1:**
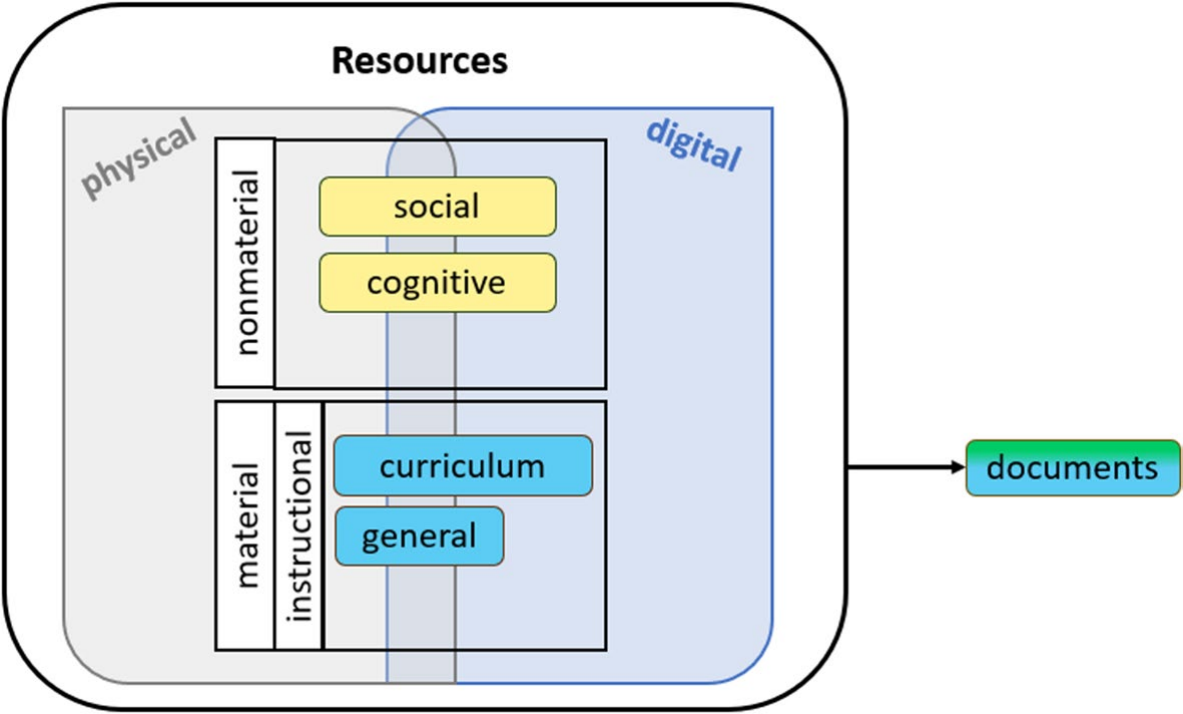
A conceptual model of resources mathematics teachers use to design and enact instruction

Resources can take physical or digital forms. According to Pepin et al. (2024), any type of resource, whether curricular, general, social, or cognitive, can exist in digital form. For example, they describe digital social resources as “formal or casual human e-based interactions” (p. 11) and cognitive digital resources as “e-based mathematical frameworks and concepts subjects work with; concepts and techniques downloadable from the web” (p. 11). Thus, while nonmaterial resources are traditionally understood as intangible, they can take digital forms in the sense that these resources are accessed and utilized through digital means. Figure 1 presents a conceptual model that integrates the various types of resources presented above, including material, nonmaterial, in both physical and digital forms. However, while some resources exist in both forms (e.g., a printed textbook vs. its e-version), others are in their nature only digital (e.g., YouTube, Wikipedia, GeoGebra), and some only physical (e.g., physical geometric solids, algebra tiles).

### Reasoning and Proving

Reasoning and proving (R&P) is essential to learning mathematics with understanding (e.g., Hanna & de Villers, 2012; National Council of Teachers of Mathematics [NCTM], 2009). Following Cai and Cirillo (2014), we consider R&P as a unified concept, where reasoning includes argument development leading to proof. Stylianides (2008a) similarly views R&P as an overarching activity that is important in the process of making sense of and establishing mathematical knowledge, encompassing four key activities: identifying a pattern, making conjectures, providing non-proof arguments, and providing proofs. Likewise, Jeannotte and Kieran (2017), proposed a model of mathematical reasoning that is constituted of two main aspects, structural and process. The latter refers to the processes related to the search for similarities and differences, or the processes related to validating, and among validating processes, one can find justifying, proving, and formal proving. In our paper, we have adopted the definition proposed by A. Stylianides (2007), who characterized proof in school mathematics as “a mathematical argument, a connected sequence of assertions for or against a mathematical claim, encompassing a set of accepted statements by the classroom community, modes of argumentation, and modes of argument representation” (p. 291). Hence, we regard R&P as accessible across all grade levels, given its capacity to adopt diverse forms of argumentation and representation contingent upon the classroom community and grade level.

## Literature Review

Several studies have highlighted challenges in teaching R&P, influenced by factors like content area, grade level (Buchbinder & McCrone, 2022), teachers’ mathematical knowledge teaching proof (Buchbinder & McCrone, 2020), and their conceptions of R&P (Herbert et al., 2015; Knuth, 2002). Prospective teachers face challenges in implementing high-level tasks related to R&P and managing students’ habits of mind (Stylianides et al., 2013). Teachers’ beliefs about the importance of proving in mathematics (Kotelawala, 2016), students’ proof capabilities (Knuth, 2002), and their instructional practices in R&P (Furinghetti & Morselli, 2011) are also significant factors. Beliefs associated with teachers’ mathematical knowledge for teaching proof, particularly knowledge of content and students, and knowledge of content and teaching (Buchbinder & McCrone, 2020), constitute teachers’ beliefs in their own ability to positively affect students’ abilities regarding R&P. This construct is in literature known as teachers’ self-efficacy or personal teaching efficacy (Liljedahl & Oesterle, 2020). In our study, we examine teachers’ self-reported confidence in R&P instruction, that is, how teachers feel about their ability to enhance students’ R&P abilities in mathematics. This reflects their perceived competence in effectively teaching and improving these specific student skills.

When it comes to methods used in studies related to mathematics teachers’ resource utilization, many are qualitative and often based on interviews (e.g., Grave & Pepin, 2015; Gueudet & Trouche, 2009; Siedel & Stylianides, 2018). However, to the best of our knowledge, only one study has particularly focused on resources for teaching R&P (Çakıroğlu et al., 2023). A survey study on curriculum resources in mathematics education conducted by Rezat (2024) covering the period from 2018 to 2023, shows that research on mathematics textbooks remains predominant, with content analysis being the most popular research area. Studies linking R&P and textbooks primarily focus on the opportunities that textbooks provide to enhance students’ R&P skills (e.g., Bergwall, 2025; Even & Silverman, 2024).

Quantitative studies on teachers’ resource use for teaching R&P are also scarce. Lepik et al. (2015) surveyed 402 mathematics teachers in grades 7 to 9 from Estonia, Finland, and Norway about their textbook use. Findings revealed that Finnish teachers primarily rely on textbooks for exercises, while those in Estonia and Norway use other resources more often. Additionally, almost 45% of teachers solely use the textbook for exercises, suggesting its broader potential is underutilized. Kock and Pepin (2019) surveyed 78 secondary school math teachers in the Netherlands on their resource choices for lesson preparation. They found national documents (such as syllabi), personal notes, colleague discussions, mathematics applications, and textbook’s websites were popular. Among digital resources, GeoGebra was the most used, followed by the digital textbook and online videos. Polly (2017) examined what curricular resources 257 American teachers in Grades 3 to 5 used to teach new mathematics standards. The findings revealed that participants were compiling various supplemental resources, primarily coming from not professionally developed Internet-based resources (26.14%) and teacher-created resources (22.75%). The reason for the compilation was the lack of a comprehensive resource that completely met the standards and the needs of all students, as well as a need to prepare their students for the high-stakes assessment adequately. Shapiro et al. (2019) surveyed 601 elementary math teachers in the USA on their online resource selection. They found teachers frequently accessed activities on “not-so-trustworthy websites” like Teachers Pay Teachers (89%), Pinterest (74%), and general Google searches (68%). Experience levels significantly influenced website preferences, with more experienced teachers favouring sites like NCTM & State Affiliates. Overall, it appears that demographic factors like years of experience, country of instruction, and grade level impact resource choices.

Pino-Fan et al. (2023) highlight teachers’ need to adeptly navigate diverse teaching contexts, utilizing both digital and material resources effectively, including textbooks, blackboards, and manipulatives. This aspect is considered a sub-competence within the'use and management of resources’ in their didactic-mathematical knowledge model. However, this competence is complex and, as Remillard (2000) and Stein et al. (1996) suggest, teachers’ decisions about what and how to implement in their classrooms to support reasoning are mediated through the use of curriculum resources.

### Summary and Research Questions

The reviewed literature has shown that teaching R&P is influenced by a variety of factors. It also highlights that while some studies focus on mathematics teachers’ resource utilization, most are qualitative and not specifically centered on R&P instruction. In response to Lin et al.’s (2012) recommendation for further investigation into teachers’ practices and beliefs related to the teaching of R&P, and considering the identified scarcity of quantitative studies on teachers’ use of resources for teaching R&P, this paper explores teachers’ resources system (Gueudet & Trouche, 2011), structured according to a general aim (planning a mathematics lesson) and the sub-aim of teaching R&P. Specifically, it addresses the following research questions:

- RQ1. What is the relationship between teachers’ use of resources in preparing mathematics lessons and for teaching R&P?
- RQ2. What is the association between teachers’ use of resources for teaching R&P, their beliefs about the importance of R&P, and their self-reported confidence in teaching R&P?
- RQ3. In what ways do demographic variables affect teachers’ use of particular resources for teaching R&P?

The documentational approach (Gueudet & Trouche, 2009, 2011) is typically used in qualitative case studies to analyse how teachers develop documents, which encompass both resources and schemes of utilization. However, as noted by Gueudet (2017), quantitative studies based on questionnaires can provide insights into the resources teachers use but not into the schemes they develop (p. 203). Given the complexity of document development and the diversity of five different cultural contexts, this study takes a different approach. Rather than investigating documents systems, which involve both resource selection and utilization schemes, we focus specifically on teachers’ resources systems within the class of situations related to lesson preparation. More precisely, we examine resources systems associated with the general aim of planning mathematics lessons and the sub-aim of planning lessons focused on R&P. By narrowing our scope to resources teachers report using, rather than how they engage with them in practice, we provide a structured, quantitative analysis of resource selection patterns across different teaching aims.

Furthermore, while we do not explicitly examine schemes of utilization, we acknowledge that teachers’ beliefs about importance of R&P and confidence in teaching R&P can be interpreted as operational invariants, which are fundamental components of utilization schemes, as they might affect how teachers engage with resources and how actively they integrate them into their R&P teaching practices. These beliefs and confidence might influence whether teachers expand their resources system (seeking out diverse resources) or limit it (relying on familiar ones).

Finally, this study considers demographic factors as potential influences on teachers’ resources systems. A teacher’s documentation work is shaped by their professional environment, institutional constraints, and access to resources (Gueudet & Trouche, 2011; Ruthven, 2009). Demographic factors such as teaching experience, educational background, school type, or other subjects taught may affect which resources teachers have access to, how they prioritize them, and the extent to which they integrate new resources into their teaching of R&P. By examining these factors, we aim to better understand how demographic characteristics possibly contribute to differences in teachers’ resources systems for teaching R&P.

## Methodology

In this section, we outline the foundational components of our research methodology, encompassing the design and deployment of a structured questionnaire, a description of our sample, national contexts, and a brief overview of the data analysis approach.

### Description of the Tool for Data Collection and Conceptualization of Resources

Data for this study were collected via an online questionnaire modelled after a survey by Kock and Pepin (2019). The questionnaire was tailored to accommodate the diverse school systems and languages of the five countries involved. To ensure clarity across national contexts, localization and cross-checking phases were conducted. Questionnaire items were translated and reviewed by MaTeK consortium members fluent in the respective languages. A pilot phase involved distributing the questionnaire to approximately 20 teachers in each country, leading to minor adjustments based on pilot survey results (Di Paola et al., 2023). The questionnaire was administered using the LimeSurvey platform.

The questionnaire was composed of 29 items divided into three sections. The first one explored teachers’ *resources system* by examining which resources they selected across different *classes of situations*: (i) preparation of lessons, (ii) refreshment or improvement of teachers’ personal knowledge in mathematics, (iii) looking for inspiration or ideas for teaching mathematics, (iv) assessment preparation, and (v) finding materials to be used in the classroom by students. Each situation was addressed through a mandatory item, with teachers rating the frequency of their resource use on a 6-point Likert scale ranging from 1 (not at all) to 6 (always). The middle points were left unlabelled to avoid overthinking and help respondents make a clear choice. The resource list, comprising 14 options (Table 1), included in brackets country-specific illustrative examples, such as well-known national online professional platforms, to facilitate consistent interpretation. An open field allowed teachers to specify any additional resources they used beyond those listed.

**Table 1:** List of resources with abbreviations

No	Resource used	No	Resource used
1	Curriculum (CR)	8	Professional periodicals/journals (J)
2	Textbooks (TB)	9	Social media (SM)
3	Teacher guides (TG)	10	Video platforms, e.g., YouTube (VP)
4	Books other than textbooks (B)	11	Online professional platforms (OPP)
5	Consultations with colleagues (CC)	12	General websites, e.g., Wikipedia (GW)
6	Self-created materials (SC)	13	Digital math apps, e.g., GeoGebra (DA)
7	Online databases for teacher-shared resources (OD)	14	Image search engines (IS)

However, our focus in this paper is solely on teachers’ reported resources for the first situation: lesson preparation. Unlike Siedel and Stylianides (2018), we focus solely on *generic* resources—broad categories such as textbooks or online professional platforms, rather than *specific* resources like a particular textbook or a specific website. This choice enables a broader comparison of resource use across different contexts, without being limited by specific resources that may vary widely. Additionally, while we acknowledge the distinction between physical and digital resource forms (Fig. 1), the questionnaire did not explicitly differentiate between resources that exist in both forms (e.g., printed vs. digital textbooks), nor did it address whether teachers combined multiple resources of the same type, as this was not a primary aim of our study.

Since the questionnaire was developed during the COVID-19 pandemic and data were collected shortly after schools reopened, there may have been an impact on teachers’ use of resources before and after the pandemic. To address this, we included two items specifically focusing on changes in resource use during the pandemic. The impact of the COVID-19 pandemic on resource use was analysed separately in Di Paola et al. (2023), however, this analysis was conducted solely on the pilot study data.

The second section of the questionnaire examined teachers’ conceptions of R&P, their resources systems associated with a sub-aim of teaching R&P, their beliefs about the importance of R&P, and self-reported confidence in teaching R&P. Questions corresponding to RQ1 and RQ2 are detailed in Table 2. Likewise, mirroring the format of the first section, participants rated their agreement with statements in items B_RP and SC_RP using a 6-point Likert scale ranging from 1 (strongly disagree) to 6 (strongly agree). In item R_RP, they chose from a list of specified resources (same as those in Table 1) or specified additional resources used for teaching concepts related to R&P.

**Table 2:** List of questionnaire items used in this study answering RQ1 and RQ2

Item number	A question posed in the questionnaire	Abbreviation used in this paper
1.1	How frequently do you use the following resources in preparing your mathematics lessons?	R_ML
2.2	In my opinion, reasoning (and for suitable grade levels, proving) should be an essential part of mathematics teaching and learning	B_RP
2.4	I know how to improve students’ reasoning and/or proving abilities in my mathematics classes	SC_RP
2.7	Which of the following resources do you usually use for teaching ideas related to reasoning and/or proving?	R_RP

The third section of the questionnaire included demographic inquiries such as age, gender, highest education level, current and past grades taught, years of teaching experience in total and in mathematics, school type, school location, and other subjects taught. Respondents had the option to provide their email addresses for potential follow-up online interviews, allowing for a deeper exploration of their R&P conceptualizations and resources interactions. These interviews thus offered a partial glimpse into teachers’ documentation work and resource utilization schemes through their reported rules of action and operational invariants. They also allowed for further exploration of used resources, whether in physical or digital form, as well as *specific* resources (Siedel & Stylianides, 2018) or instances where multiple resources of the same type were combined (e.g., several textbooks or professional platforms), including the reasons behind these choices (Çakıroğlu et al., 2023; Høynes & Kohanová, in press).

### Participants

Data collection spanned from April to September 2022 across all five countries. The questionnaire was distributed to schools via email invitations sent directly to principals. In parallel, we shared the link to the questionnaire on social media pages for mathematics teachers to reach them directly. This approach led to 1646 responses (not all of which were completed), with the response rates per country shown in Table 3. For the analysis, responses from teachers who completed all relevant items for the three research questions were used, yielding between 790 and 836 responses depending on completion rates. For each research question, responses with any missing relevant items were removed from the corresponding analysis.

**Table 3:** Number of complete responses included in the analysis and rate of return

Country	Number of complete responses	Rate of return
Czech Republic	125	45.45%
Italy	105	41.18%
Norway	143	53.76%
Slovakia	344	81.13%
Turkey	119	36.73%
Total	836	-

The sample comprised 624 women (74.6%) and 190 men (22.7%), with 22 participants either leaving the item unanswered or selecting the option ‘prefer not to say’. Approximately 6.82% of respondents taught in private schools. The majority of participants were experienced mathematics teachers with master’s degrees, reflecting the current requirements in most countries. The sample exhibited a balanced distribution across different school levels: Level 1 (grades 1–5), Level 2 (grades 6–9), and Level 3 (grades 10–13). See Table 4 for a summary of participants’ teaching experience, highest degree attained, and school levels taught at the time of data collection.

**Table 4:** An overview of participants’ teaching background, qualifications, and school levels

Math Teaching Experience			The highest degree attained			School levels		
	N	%		N	%		N	%
*1 st year*	32	3.8	*High School*	27	3.2	*Level 1*	172	20.6
*2–5 years*	109	13	*Bachelors*	210	25.1	*Level 2*	227	27.2
*6–10 years*	156	18.7	*Masters*	551	65.9	*Level 3*	156	18.7
*11–15 years*	120	14.4	*PhD*	41	4.9	*Level 1&2*	187	22.4
*16–20 years*	120	14.4	*Not specified*	7	0.84	*Level 2&3*	78	9.3
*Over 20 years*	295	35.3				*All levels*	5	0.6
*Not specified*	4	0.48				*Not specified*	11	1.3
Total	836	100	Total	836	100	Total	836	100

### National Contexts

The subsequent sections offer information regarding national contexts concerning available resources, school systems, and R&P across the five countries involved. Each country possesses centrally determined national curricula, all addressing R&P to varying extents.

The Czech national curriculum for primary education (grades 1–9) includes R&P, but only briefly and in connection to problem-solving and geometry. In the general part of the grades 10–13 curriculum, broader competencies related to R&P, like the development of logical thinking, hypothesis testing, and critical evaluation, are mentioned. Additionally, there is a specific theme titled “Argumentation and verification” that is intended to be taught.

The theme of R&P, expressed in the Italian mathematics curriculum for grades 1–13, is generally presented as crucial. For grades 1–8, it refers to R&P in terms of starting point to mathematical proving and as a process of argumentation, logical thinking, hypothesis testing, and critical evaluation, in connection to problem solving. In grades 9–13, “Arguing, Conjecturing and Proving” is one of the three transversal nuclei, called “process”.

In Norway, “Reasoning and argumentation” is one of six core elements in the mathematics curriculum for grades 1–13, highlighting mainly processes related to explanation, justification, and validation. Conjecturing is mentioned as well, but under a different core element. On the other hand, proving is not explicitly included among competency goals until grade 11.

In Slovakia, “Mathematical language, communication and argumentation” is one of the three core elements in the mathematics curriculum for grades 1–9, with “to think critically and argue” as one of the six main goals of the subject. For grades 10–13, R&P is mentioned in general goals and also as one of five themes to be taught, called “Logic, justification, and proofs”.

In Turkey, Reasoning and proof are mentioned in the specific goals of the grades 1–8 mathematics curriculum as “(Pupils) will be able to easily express their own thoughts and reasoning in the problem-solving process and will be able to see the gaps or deficiencies in the mathematical reasoning of others.” (Ministry of National Education [MoNE], 2018, p. 9). The grades 9–12 mathematics curriculum mentions it in more general terms in its goals. In addition, in grade 9, students are expected to learn the term ‘proof’ under the topic of logic.

In all five countries, primary school teachers are not subject specialists. In Norway, these teachers handle grades 1–7; however, in most schools, mathematics is taught by subject specialists from grade 5 onwards. The pattern is similar in the remaining countries, where mathematics is taught by subject specialists beginning in grade 5 (Slovakia and Turkey) or 6 (Czech Republic and Italy).

In all five countries, most classrooms are equipped with digital tools, including computers or tablets and interactive whiteboards. Norway stands out with widespread access to tablets or computers for students in mathematics classes. Except for Turkey, schools or teachers can select mathematics textbooks freely. Slovakia and the Czech Republic follow a centralized list, while Italy and Norway lack such regulation. Overall, teachers in these countries have the flexibility to integrate supplementary resources, including alternative textbooks.

### Data Analysis Tools

We first summarize that the answers to items R_ML, B_RP, and SC_RP are integer values 1–6 and the answers to R_RP are binary values 0/1 (No/Yes). To answer RQ1, the observed association between the tendency to use a given resource when teaching R&P (R_RP) and its overall usage in preparing mathematics lessons (R_ML) was quantified by calculating Pearson’s correlation coefficient between the answers to these questions for each resource. The significance of the association was then tested for each resource. Because the data were collected from multiple countries, the Cochran-Mantel–Haenszel tests (see Agresti, 2013, Sect. 6.4) were used, which take the stratification by country into account. The integer values 1–6 of R_RP are treated as categorical in the Cochran-Mantel–Haenszel tests, which means that a general association, not necessarily just correlation of numeric variables, was tested.

The analysis performed for RQ2 was analogous to that for the first question. Pearson’s correlation coefficients between R_RP and (i) teachers’ beliefs about the importance of R&P (item B_RP), (ii) teachers’ self-reported confidence in teaching R&P (item SC_RP), were calculated. This was followed by applying the Cochran-Mantel–Haenszel tests for stratified data to test the significance of the associations.

Finally, to answer RQ3, a series of logistic regressions were performed to examine the dependence of R_RP on the demographical characteristics of the teachers per resource, because they consider the effects of multiple variables simultaneously. As such, the effect of one characteristic corrected for the effects of the other characteristics could be tested. Specifically, the independent variable in each regression was R_RP for the given resource, and the demographical characteristics were the dependent variables. The significance of the association between R_RP and the teachers’ characteristics was analysed by submodel testing in the logistic regression via the chi-squared test.

## Results

The findings of this study are presented in three sections, each corresponding to one of the three research questions posed.

### Resources used in Preparing Mathematics Lessons and for Teaching R&P (RQ1)

Before examining the relationship between resource use for the general aim of planning mathematics lessons and the sub-aim of planning lessons focused on R&P, we first provide an overview of the overall usage tendencies. Table 5 summarizes the average frequency of use for each resource in lesson preparation (R_ML) and the proportion of teachers who reported using each resource for teaching R&P (R_RP). For example, textbooks (TB), books other than textbooks (B), and self-created (SC) materials were frequently selected within teachers’ resources systems for lesson preparation, and a large proportion of teachers also reported using these resources within the sub-aim of teaching R&P. In contrast, fewer teachers selected resources like consultations with colleagues (CC) or online databases for teacher-shared resources (OD) for R&P instruction. This overview highlights the structure of teachers’ resources systems, illustrating how different resources are prioritized for lesson preparation and R&P instruction, providing important context for the subsequent correlation analysis.

**Table 5:** Resource usage tendency and Pearson’s correlation coefficients

Resource	R_ML (avg)	R_RP (%)	R_RP correlation with		
			R_ML	B_RP	SC_RP
CR (Curriculum)	3.85	17.58	**0.30** (< 0.001)	0.04 (0.07)	0.08 (0.60)
TB (Textbooks)	4.39	56.34	**0.32** (< 0.001)	−0.02 (0.65)	−0.04 (0.84)
TG (Teacher guides)	3.49	39.83	**0.37** (< 0.001)	−0.01 (0.85)	−0.03 (0.42)
B (Books other than textbooks)	3.96	50.84	**0.31** (< 0.001)	**0.14** (0.02)	**0.14** (0.03)
CC (Consultations with colleagues)	3.93	35.65	**0.30** (< 0.001)	0.04 (0.65)	0.01 (0.99)
SC (Self-created materials)	4.44	50.72	**0.27** (< 0.001)	**0.12** (0.003)	**0.16** (< 0.001)
OD (Online databases for teacher-shared resources)	3.97	35.17	**0.32** (< 0.001)	−0.05 (0.08)	−0.02 (0.33)
J (Professional periodicals/journals)	2.24	11.84	**0.39** (< 0.001)	0.15 (0.12)	**0.20** (0.004)
SM (Social media)	2.63	15.07	**0.39** (< 0.001)	0.12 (0.37)	0.09 (0.75)
VP (Video platforms)	3.17	33.01	**0.30** (< 0.001)	0.07 (0.19)	0.01 (0.57)
OPP (Online professional platforms)	3.03	23.92	**0.40** (< 0.001)	**0.17** (0.03)	0.11 (0.33)
GW (General websites)	2.64	16.27	**0.23** (< 0.001)	0.09 (0.65)	0.05 (0.80)
DA (Digital math apps)	3.17	25.84	**0.43** (< 0.001)	0.12 (0.29)	**0.15** (0.01)
IS (Image search engines)	3.21	14.35	**0.28** (< 0.001)	0.06 (0.89)	0.14 (0.20)

Table 5 also presents correlation coefficients between R_RP and R_ML for all 14 resources, ranging from 0.23 to 0.43, and p-values of the significance tests of associations in parentheses. All associations are statistically significant (p < 0.05) and the coefficients are therefore highlighted in bold. These results indicate that teachers who use a resource more frequently for preparing mathematics lessons also tend to use it more for teaching R&P.

**Fig. 2:**
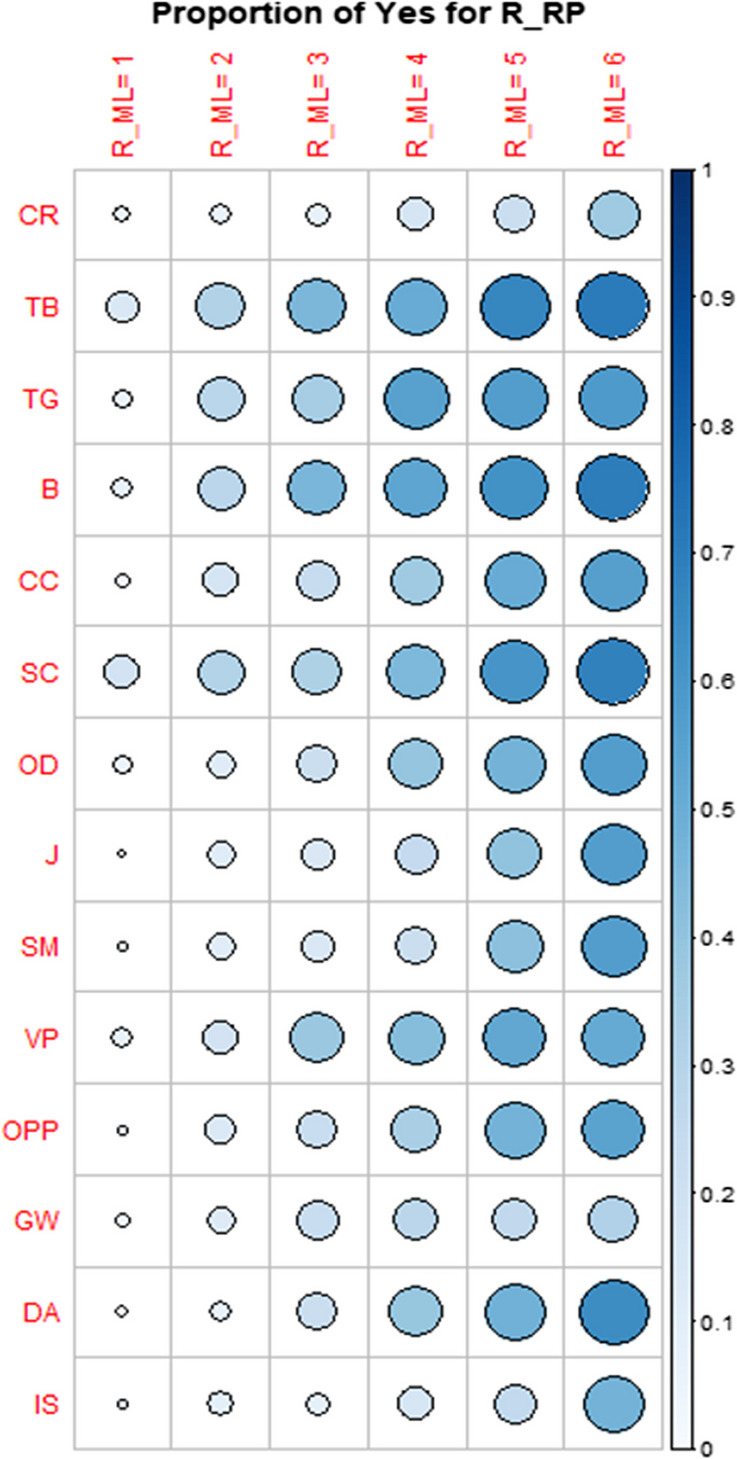
The association between using different resources for teaching R&P and their use in preparing mathematics lessons

As shown in Table 5, the weakest correlation between R_RP and R_ML is observed with General websites (GW). We can also visually observe the weak positive association in Fig. 2 in the row “GW”, where the size of the circle is stagnating. Larger and darker circles represent larger proportions of respondents who use the resource for teaching R&P. In other words, the use of GW in preparing mathematics lessons is not strongly associated with using GW for teaching R&P.

Based on the results presented in Table 5 (column showing the correlations between R_RP and R_ML) and Fig. 2, the largest positive associations were observed in four cases when the same resource was used for different purposes. These four cases include Professional periodicals/journals (J), Social media (SM), Online professional platforms (OPP), and Digital math apps (DA). For example, suppose the teacher never uses DA in preparing mathematics lessons (Fig. 2, column R_ML = 1). In that case, this teacher almost certainly answers “No” to questions about the use of the same resource for teaching R&P. If s/he always uses this resource in preparing mathematics lessons (Fig. 2, column R_ML = 6), s/he tends to answer Yes more often than No to the question concerning using this resource for teaching R&P.

Additionally, among the most frequently used resources for lesson planning, self-created materials (SC) have the highest average frequency of use (4.44; about 77% of teachers report frequent use), yet “only” about half of teachers reported using them for R&P instruction, with a relatively weak correlation (r = 0.27). Similarly, textbooks (TB) are widely used (4.39; about 70% report frequent use), but just over half of teachers select them for R&P (r = 0.32). In contrast, as already mentioned, DA are used less frequently (3.17) but have the strongest correlation (r = 0.43) and are chosen for R&P by about a quarter of teachers. These differences suggest that while some resources are central to teachers’ resources systems and are widely integrated into documentation work for general lesson planning, their transferability to R&P instruction is not straightforward.

In summary, RQ1 findings suggest a link between resource usage in lesson planning and teaching R&P: teachers who use a resource in lesson preparation tend to use it more for R&P instruction. However, while the associations are statistically significant, the corresponding correlations are only moderate, indicating similarity but not complete alignment in resource usage. Furthermore, association strength varies across resources, as evident in Table 5’s diverse correlation coefficients. This suggests that teachers’ resources systems integrate some resources consistently across teaching aims, while others are more specialized or selectively adapted for R&P.

### Resources for Teaching R&P, Teachers’ Beliefs and their Self-Confidence in Teaching R&P (RQ2)

The significance of the association between resource use for teaching R&P and teachers’ beliefs about R&P importance (B_RP) varies. Only three resources—Books other than textbooks (B), Self-created materials (SC), and Online professional platforms (OPP)—show statistically significant associations (p < 0.05, see Table 5). However, these associations are weak (r: 0.14, 0.12, and 0.17, respectively), suggesting a weak relationship between resource use and teachers’ beliefs about R&P importance. This pattern indicates that teachers with stronger beliefs about the R&P importance are likely drawing on specific *operational invariants* when using professionally prepared resources (B, OPP) and their self-created curriculum materials (SC) to meet the diverse needs of their classrooms.

On the other hand, teachers’ self-reported confidence in teaching R&P (SC_RP) showed significant associations with a broader range of resources, including Books other than textbooks (B), Self-created materials (SC), Professional periodicals/journals (J), and Digital math apps (DA). However, Pearson’s correlation coefficients (r: 0.14 to 0.20; Table 5, column SC_RP) indicate a weak association between R_RP and SC_RP responses. Higher self-reported confidence in teaching R&P is linked to increased use of professional digital and physical resources (B, J, DA), as well as the utilization of self-created curriculum materials (SC) for classroom instruction.

**Fig. 3:**
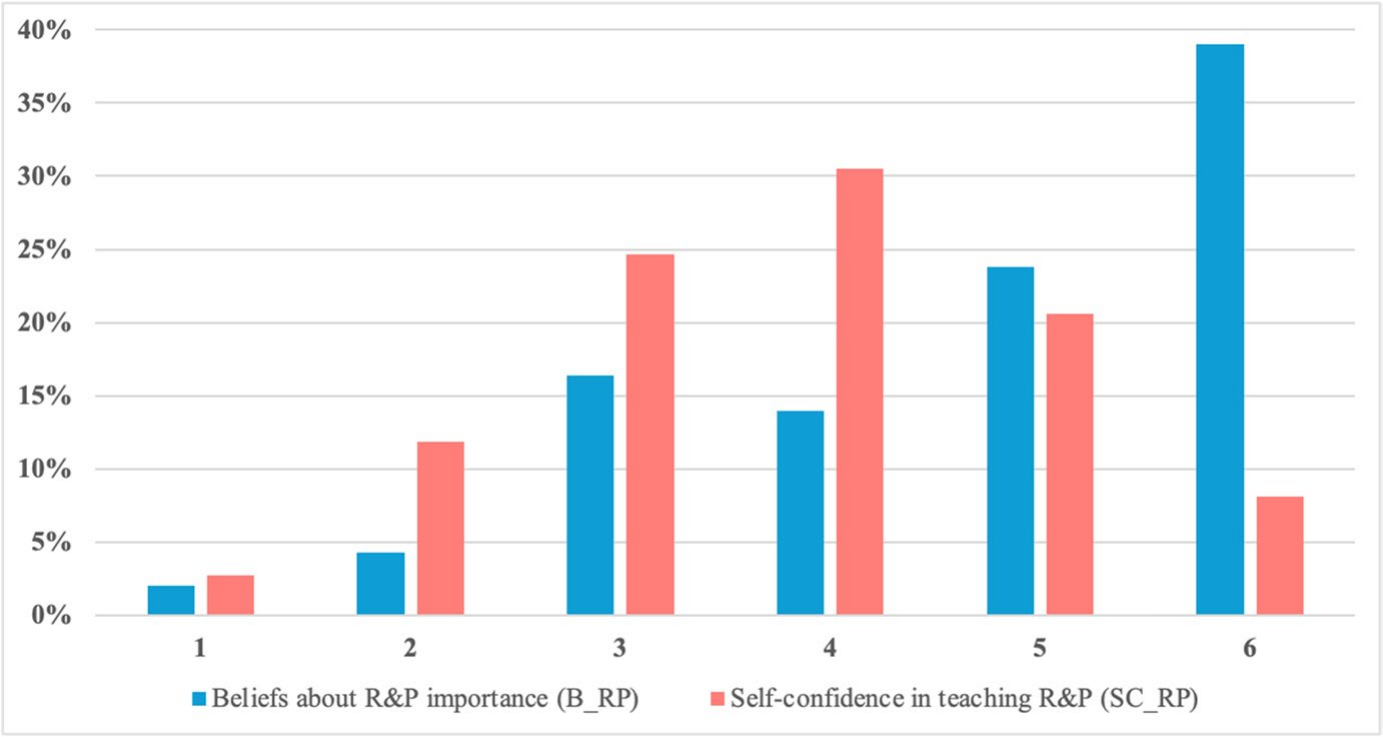
Percentage of teachers rating their beliefs about R&P importance and their confidence in teaching R&P

Additionally, the analysis of teachers’ responses to the B_RP and SC_RP items (on a 6-point Likert scale, 1 = strongly disagree, 6 = strongly agree) revealed that less than 3% of respondents selected the lowest value for both items (see Fig. 3). However, there is a notable disparity in the selection of the highest possible value on the scale. Specifically, 39% of respondents chose the maximum value when rating the importance of R&P in mathematics teaching, whereas only about 8% selected the highest value when self-assessing their confidence in their R&P instructional skills. This suggests that while teachers recognize the importance of R&P, they perceive themselves as less confident in their ability to teach it effectively, as reflected in their lower self-assessment ratings.

### Resources for Teaching R&P and Demographic Variables (RQ3)

The association between teachers’ use of particular resources for teaching R&P and the demographics was analysed via logistic regression. The effects of age, teaching experience, mathematics teaching experience, and teaching other subjects than mathematics were found by the chi-squared test not to be significant (p > 0.05) for all or nearly all resources; they were therefore removed from the models. The goodness of fit of the logistic regressions was tested using the Hosmer–Lemeshow test (see Faraway, 2016, Sect. 2.6); the results are given in Table 6. The tests confirmed the quality of all fits (note that for the Hosmer–Lemeshow test, p < 0.05 signifies a lack of fit and p > 0.05 suggests a good fit). The significance of the remaining characteristics was again tested by the chi-squared test, with relatively few factors being significant (see Table 7). A further chi-squared test was used to identify the pairs of school levels with significantly different effects; again, see Table 7.

**Table 6:** Hosmer–Lemeshow tests

Resource	p-value	Resource	p-value
CR (curriculum)	0.183	J (professional periodicals/journals)	0.053
TB (textbook)	0.076	SM (social media)	0.874
TG (teacher guides)	0.947	VP (video platforms)	0.357
B (books other than textbooks)	0.820	OPP (online professional platforms)	0.956
C (consultation with colleagues)	0.539	GW (General websites)	0.884
SC (self-created materials)	0.977	DA (digital math apps)	0.835
OD (online databases for teacher-shared resources)	0.196	IS (image search engines)	0.714

**Table 7:** Factors with significant effects on resource usage for teaching R&P

Resource	Factors	Resource	Factors
CR (curriculum)	Country, School level (1 vs. 2, 1 vs. 3)	J (professional periodicals/journals)	Country
TB (textbook)	School level (1 vs. 3)	SM (social media)	Country
TG (teacher guides)	Country, School level (1 vs. 2, 1 vs. 3)	VP (video platforms)	Country, Private school
B (books other than textbooks)	Country, School level (1 vs. 2)	OPP (online professional platforms)	Country, Private school
C (consultation with colleagues)	Country	GW (General websites)	Country, Gender, Private school
SC (self-created materials)	School level (1 vs. 3, 2 vs. 3)	DA (digital math apps)	Country, School level (1 vs. 2, 1 vs. 3, 2 vs. 3), Private school
OD (online databases for teacher-shared resources)	Country, School level (1 vs. 3, 2 vs. 3), Gender	IS (image search engines)	Country, Private school

**Fig. 4:**
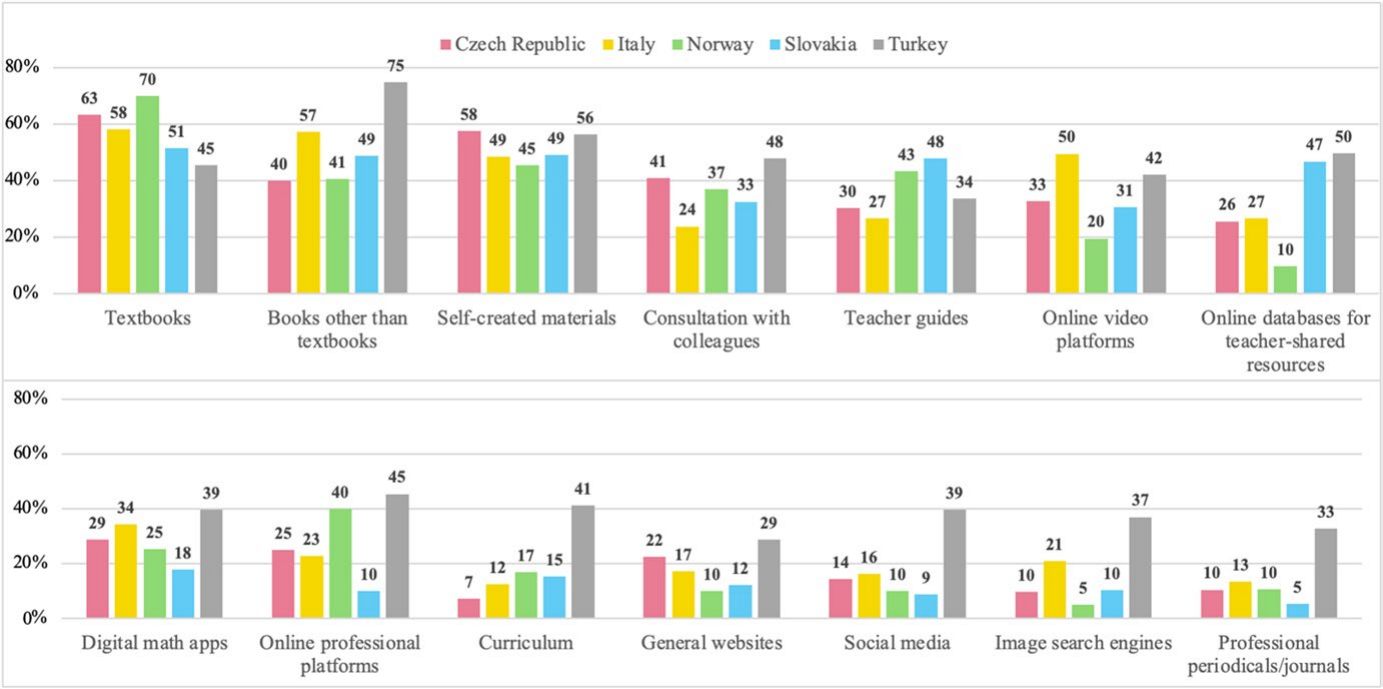
Percentage of teachers using particular resources for teaching R&P in each country (values rounded to the nearest integer)

Country was the factor that appeared most important, with only two specific resources not impacted, namely textbooks (TB) and self-created materials (SC). Despite geographical differences, teachers consistently rely on TB and SC across all countries studied. This intriguing finding prompted a closer descriptive examination of TB and SC across countries. Specifically:

- TB are the predominant resource for teaching R&P in all countries except Turkey, with usage rates ranging from 51.45% in Slovakia to 69.93% in Norway (Fig. 4).
- SC rank as the second most commonly used resource for teaching R&P in all countries except Italy, with usage rates ranging from 45.45% in Norway to 57.60% in the Czech Republic.

School level significantly influences the use of several resources (CR, TB, TG, B, SC, OD, DA), with the country of instruction also being a factor for CR, TG, B, OD, and DA. Specifically:

- Teachers at upper secondary schools (grades 10–13) use TB, SC, and DA more frequently, but fewer utilize OD compared to lower-grade teachers.
- Primary school teachers (grades 1–5) rely more on TG for teaching R&P than teachers in higher grades.
- Although differences in CR and B usage between levels are significant, we lack a clear interpretation, possibly due to teachers teaching multiple levels or the relatively small differences observed.

Another finding relates to digital resources (VP, OPP, GW, DA, and IS), with private schools showing a significant preference for these resources compared to public schools. Gender differences are observed in two resources: OD, favoured by more women, particularly in Italy, and GW, more commonly used by men, especially among Czech and Norwegian teachers.

## Discussion

In our study, we investigated the resources systems of mathematics teachers across five European countries, mainly focusing on their use of resources for preparing and teaching lessons related to R&P. Using the Documentational Approach to Didactics (Trouche et al., 2020) as a framework and the conceptual model presented in Fig. 1, we explored patterns in resource selection within teachers’ resources systems. Additionally, we examined how teachers’ resource selection is influenced by various demographic factors, teachers’ beliefs, and self-reported confidence. In the subsequent sections, we discuss the findings related to each research question individually, highlighting how the DAD framework guided our interpretation of the results.

The analysis of the relationship studied in RQ1, namely between teachers’ use of resources in preparing mathematics lessons (R_ML) and for teaching R&P (R_RP), revealed several noteworthy findings. Although statistically significant, the positive associations between resource use for the two mentioned aims varied in strength, with correlation coefficients ranging from 0.23 to 0.43. These coefficients suggest a moderate level of association. However, it’s essential to recognize that these correlations solely reflect linear relationships and may not encompass all influencing factors or *operational invariants*. Among the resources examined, General websites (GW) exhibited the weakest correlation, suggesting a less consistent relationship between their use in lesson preparation and teaching R&P. Teachers might perceive GW (e.g. Wikipedia) as less effective for teaching R&P because they’re not tailored for curriculum enactment and often lack suitable content and formats for school mathematics, which is a limitation of general (non-curricular) resources. In contrast, Professional periodicals/journals (J), Social media (SM), Online professional platforms (OPP), and Digital math apps (DA) demonstrated the strongest correlations, indicating a more pronounced association between resource use for both aims. The use of resources like J and OPP, which are professionally prepared, indicates teachers actively seek new approaches to teaching, update their knowledge, and try to apply research results connected to enhance their teaching practice related to R&P, as this is a relatively new topic recently appearing in curricula of involved countries. However, resources J and OPP are not the most common resources for teaching ideas related to R&P among teachers in our sample (see Fig. 4). This finding aligns with the results of Polly’s (2017) and Shapiro et al.’s (2019) studies, which revealed that teachers often use resources that are not professionally developed.

Similarly, neither social media (SM) is among the most used ones for teaching ideas related to R&P in our sample. This might be because teachers are not fully aware that SM can be valuable tools for lesson planning, as Kock and Pepin (2019) suggested. The utilization of Digital math apps (DA) is also not very common but consistent across the sample (see Figs. 2 and 4). Teachers’ use of DA can be caused by its range of uses, providing new perspectives on pupils’ understanding development, and enhancement of student learning outcomes (Drijvers et al., 2016). We hypothesize that some teachers recognize the potential of certain DA (like GeoGebra) to facilitate R&P tasks, as they can serve as a tool to build visual proofs or for conjecturing and developing conviction (Zengin, 2017). Additionally, many teachers participate in professional development courses across all participating countries, where the features of DA (e.g., GeoGebra, Phet) are often presented.

In the case of the most frequently used resources for lesson planning (SC and TB), where the associations between R_ML and R_RP are relatively weak (Table 5), the issue appears to be that teachers may not yet have sufficient self-created materials (SC), or do not always find what they “need” for teaching R&P in textbooks (TB). The latter issue, concerning TB, was observed across all five countries (Çakıroğlu et al., 2023). In other words, while TB may serve as a starting point, teachers often supplement them with additional resources to better support R&P instruction, which may explain the relatively weak association observed. Similarly, although SC are widely used for general lesson planning, they may not yet be sufficiently developed or adapted for the specific demands of R&P, which could account for the even weaker association in their case.

In summary, while the significant associations underscore the interconnectedness between resource use in lesson preparation and teaching R&P, the moderate correlation coefficients suggest a nuanced relationship and indicate the complexity of teaching. Teachers’ resource selection for R&P instruction may be influenced by *operational invariants* beyond their use in mathematics lesson preparation, like teachers’ conceptions of R&P (Herbert et al., 2015; Knuth, 2002), teachers’ mathematical knowledge for teaching proof (Buchbinder & McCrone, 2020) or mathematical knowledge for teaching related to reasoning, i.e. task selection, teacher questioning, and assessment, as Davidson et al. (2019) concluded. The varying correlation strengths among different resources suggest that teachers’ resources systems are structured yet adaptable, integrating both widely used curriculum resources (e.g., self-created materials, textbooks) and more specialized resources (e.g., digital applications). This implies that certain resources may better support teaching R&P than others, emphasizing the need for careful resource selection to align with instructional goals—an important competence as highlighted by Pino-Fan et al. (2023).

The RQ2 aimed to uncover links between teachers’ resource utilization for teaching R&P, their beliefs about R&P importance, and their self-reported confidence in R&P instruction. Results revealed statistically significant yet weak associations between the use of some resources (B, SC, OPP) and beliefs about R&P importance. This suggests that while teachers with stronger beliefs may use these resources more, other unexplored factors or *operational invariants* may also shape how various resources are integrated into their resources systems for R&P instruction. This finding underscores the multifaceted nature of teacher practices and beliefs in the domain of R&P instruction.

Similarly, the significant associations between resource use (B, SC, J, DA) and self-reported confidence in teaching R&P suggest that teachers with higher ratings of own confidence may indeed utilize a broader range of resources, indicating a larger ‘pool of possibilities’ (Siedel & Stylianides, 2018). However, the relatively low correlation coefficients indicate that self-reported confidence may also be influenced by other factors, such as *operational invariants*, like subject matter knowledge (Buchbinder & McCrone, 2020), and competence in the ‘use and management of resources’ (Pino-Fan et al., 2023), and potentially by contextual factors like the type of school and the type of students, as suggested by Furinghetti and Morselli (2011).

Descriptive statistics (Fig. 3) reveal that while about 39% of teachers view R&P as essential, only about 8% feel strongly confident in their ability to enhance students’ R&P skills. This indicates a gap between perceived importance and self-assessed teaching capability, which may be linked to limited classroom experience (as R&P is a relatively new instructional focus across the studied countries), perceived students’ difficulties (Furinghetti & Morselli, 2011), inadequate training (Buchbinder & McCrone, 2023) or insufficient resources or professional support (Stylianides et al., 2017). The latter was confirmed by Çakıroğlu et al. (2023) and Høynes and Kohanová (in press), who analysed the interviews with selected teachers forming the sample of this study. Therefore, we believe that targeted professional development is necessary to support teachers in effectively integrating R&P into their instruction.

Overall, the results highlight the need for a comprehensive understanding of the factors that shape teachers’ practices and beliefs regarding R&P instruction. Moreover, in our study, we noticed both positive and negative weak correlations, underscoring the importance of delving deeper into the underlying phenomena. Future research, adopting qualitative methods, could explore additional *operational invariants* and variables that may influence the relationship between resource use, beliefs, and self-reported confidence in teaching R&P. This could provide deeper insights into teachers’ documentation work, which is essential for developing effective strategies for supporting them in enhancing their R&P instruction.

The findings regarding RQ3 illuminate how demographic factors shape teachers’ resources systems associated with R&P instruction, with country being a significant determinant for most resources. Notably, textbooks (TB) and self-created materials (SC) demonstrated consistent utilization patterns across national contexts, suggesting that these curriculum resources form a core part of teachers’ resources systems, regardless of the country. TB emerged as the primary resource for R&P instruction in all countries except Turkey, aligning with Remillard and Heck’s (2014) observation that textbooks (and curriculum guides) are the most common form of instructional materials used throughout the world (p. 707). Our results suggest that TB are used by teachers no matter what topic/construct is at the core of the lesson. The observed differences in TB utilization patterns between school levels, in favour for upper secondary grades, suggest that teachers adapt their resource selection and integration to align with the increasing complexity of R&P tasks at this level. Since proof predominantly occurs in higher grades across all five countries, as outlined in the National Contexts section, teachers may rely more heavily on TB to address these complexities. Moreover, the widespread use of TB for teaching R&P across four countries suggests their perceived accessibility in introducing R&P concepts in the classroom. This consistency may stem from factors such as institutional preferences, alignment with curriculum standards, or the availability of textbooks, as detailed in the National Contexts section.

Another not country dependent curriculum resource is teachers’ self-created materials (SC). SC are consistently utilized as the second most preferred resource for teaching R&P across all countries studied, except for Italy, where they rank fourth. We identified a difference in utilization patterns between school levels, again in favour for upper secondary school teachers. We hypothesize that since teachers do not always find what they need in the most commonly used resource, namely TB (Çakıroğlu et al., 2023), they prepare their own *documents*, irrespective of their country of instruction. In some countries, SC seem to be often supplemented by materials created and shared by fellow teachers in online databases (OD), a finding similar to the one of Polly (2017). In addition, teachers use worksheets or interactive materials available in digital math apps (DA), such as GeoGebra or Phet. This preference for utilizing SC and supplementary digital resources from OD and DA is notably more pronounced also among upper secondary school teachers, suggesting a distinct pattern in how teachers structure their resources systems at this educational level. However, the use of OD and DA, but also other digital resources, like video platforms (VP), general websites (GW), image search engines (IS), and online professional platforms (OPP), is country dependent, suggesting not consistent patterns across countries. In the case of OPP, the inconsistency is very likely caused due to teachers’ awareness of the existence of such platforms, the complexity of provided information/materials, or trust in the institution managing or creating materials offered on these platforms. The utilization of the other mentioned digital resources could also differ due to the globalization of the curriculum publishing industry and availability in terms of language, as pointed out by Remillard (2019).

In our analysis, a significant contrast emerged in the use of digital resources, including VP, OPP, GW, DA, and IS, between teachers from private and public schools, with teachers from private institutions demonstrating higher usage rates. Notably, our dataset included private school teachers only from Turkey and the Czech Republic, where facilities are typically better equipped compared to their public counterparts. This suggests that better access to digital tools in private schools may shape teachers’ documentation work, influencing how they structure their resources systems for teaching R&P.

The variation in curriculum (CR) utilization for teaching R&P is noteworthy, particularly considering its dependency on the country. This discrepancy may be attributed to differences in the level of detail within national curricula. For instance, Turkey’s curriculum, reported to be used for R&P instruction by about 41% of Turkish teachers, includes specific learning goals with exemplification tasks. In contrast, the Czech Republic’s national curriculum, reported by only 7% of Czech teachers, lists necessary concepts without explicit instructional guidance. Such distinctions likely stem from diverse cultural traditions across the studied contexts (Pepin & Haggarty, 2001), potentially shaping perceptions of curriculum utility in lesson preparation and R&P enactment. The results concerning CR utilization could also carry political implications for educational systems, extending beyond the countries studied.

Finally, neither the age of teachers nor their years of experience are significant factors determining which resources teachers utilize, contradicting the results of Shapiro et al. (2019). Given the relatively recent emphasis on R&P within national curricula in the studied countries, and the difficulty of recognizing and let alone teaching R&P (Herbert & Williams, 2023), teachers may still be adapting to incorporating R&P into their instructional practices. This ongoing adaptation may reduce the influence of factors such as age and experience on resource selection.

## Conclusions

The findings reveal a significant yet modest positive association between the use of resources in preparing mathematics lessons and for teaching R&P. Additionally, in our sample, teachers’ beliefs on the importance of R&P and self-reported confidence in teaching R&P played only a minor role in shaping their resource systems associated with teaching R&P. We identified a weak but statistically significant association regarding the use of professional instructional resources and self-created materials. Factors influencing resource use varied, with country being a prominent determinant across most cases. Grade level emerged as an influencing factor for traditional resources and school type for digital resources.

These findings can be better understood through the lens of the underlying model depicted in Fig. 1, which categorizes resources as material (curriculum and general) and nonmaterial (social and cognitive), and further distinguishes between physical and digital forms. Our study suggests that curriculum resources like textbooks (TB) and self-created materials (SC) are consistently utilized across different cultural contexts, indicating their central role in teachers’ documentation work. Conducting similar research in other countries could be beneficial, as teacher behaviour differs across nations for almost all resource types. However, this discrepancy is less apparent for textbooks and self-created materials, suggesting the importance of continuing cross-national studies on textbooks use and on the creation of *documents*, for teaching ideas related to R&P.

The weak association identified in RQ2 could be a starting point for the realization of in-depth case studies of individual teachers to gain a deeper understanding of the factors and *operational invariants* influencing resource selection and integration, as well as their beliefs regarding R&P. Also, cross-cultural comparison across multiple countries exploring how cultural differences impact teachers’ resources systems and beliefs about R&P could shed more light on the phenomenon. Moreover, implementation and evaluation of professional development interventions aimed at enhancing teachers’ resource selection skills and self-confidence in teaching R&P could contribute to more effective documentation work.

Overall, these results could be used by educational policymakers and curriculum developers to facilitate more targeted and supportive reforms. Additionally, this study’s findings may enrich teacher training programs, equipping educators with the necessary knowledge to teach R&P. Follow-up research in the above-mentioned areas could further enhance our understanding of teachers’ resources systems and their documentation work and contribute to the development of effective strategies for promoting high-quality mathematics instruction.

### Limitations of the Study

We recognize several limitations in our study, such as the sample size and the uneven distribution of respondents among countries. While these particular issues were addressed by using specialized statistical tests, other limitations remain. Although linguistic comparability was taken into account and statistical methods controlled for country effects, cultural response styles in the use of Likert scales may still have influenced cross-national comparability, which is an acknowledged challenge in international comparative research (He & van de Vijver, 2012). Another limitation is the variability in teachers’ conceptions of R&P (Herbert et al., 2015; Knuth, 2002). Understanding these might be the key element in exploring their practices in resource utilization. In other words, supplementing our quantitative findings with qualitative data, such as interviews or content analysis of the open question regarding conceptualization of the R&P that was part of our questionnaire, would offer a richer perspective and deeper understanding on teachers’ practices regarding utilization of resources for teaching R&P. Although we have the data available, this was beyond the scope of the current paper, and we acknowledge that this remains a limitation of it. On the other hand, our goal was to contribute to the limited number of quantitative studies related to teachers’ use of resources for teaching R&P, which we believe is a crucial step in advancing our understanding of teaching R&P in the times of implementation of curriculum reforms. Future research could also benefit from incorporating measures related to self-reported confidence in R&P instruction and beliefs about R&P. For instance, adopting a more nuanced approach, such as multivariable analysis, could help control for demographic factors and explore their relationship with teachers’ self-reported confidence in R&P instruction. While our current research questions do not focus on this aspect, we believe that discussing the interaction between demographic variables and self-reported confidence would contribute to a deeper understanding of the complexities involved in teachers’ resource use for R&P instruction.

Lastly, the continued growth in the use of digital resources will likely influence the evolution of teachers’ resources systems. However, in the questionnaire, we did not distinguish between the physical and digital forms of resources, such as textbooks, which represents a limitation in our ability to fully analyse the impact of digital versus physical resources within the underlying model (Fig. 1). Additionally, the questionnaire did not enable us to determine whether certain resources (e.g., professional periodicals/journals) were used primarily as cognitive resources—supporting teachers’ conceptual understanding or pedagogical frames, or as curriculum resources—offering concrete materials like tasks or lesson plans. Finally, although teachers had the option to list additional resources beyond the 14 predefined ones, most left this field blank, and these responses were not included in the analysis. This may suggest that most respondents found the predefined resources sufficient, though some may not have considered additional resources, potentially limiting our understanding of their full resources systems.

Revisiting this study in the future could reveal how resources systems have adapted to changes in resource availability, particularly with the expansion of digital and AI-driven resources (e.g., ChatGPT), which were not available during our data collection. As new elements in teachers’ resources systems, these technologies might reshape the educational landscape, influencing how teachers design and enact mathematics lessons and R&P instruction. Future research should consider how these developments influence teachers’ *operational invariants* and their subsequent resource selection and integration, offering a deeper understanding of the ongoing transformation in instructional practices. By linking these findings to the underlying model (Fig. 1), we can better understand the roles of different types of resources in shaping teachers’ instructional strategies.

## Data Availability

The data that support the findings of this study are available on request from the corresponding author.
